# Evaluating the Margins of Breast Cancer Tumors by Using Digital Breast Tomosynthesis with Deep Learning: A Preliminary Assessment

**DOI:** 10.3390/diagnostics14101032

**Published:** 2024-05-16

**Authors:** Wei-Chung Shia, Yu-Hsun Kuo, Fang-Rong Hsu, Joseph Lin, Wen-Pei Wu, Hwa-Koon Wu, Wei-Cheng Yeh, Dar-Ren Chen

**Affiliations:** 1 Molecular Medicine Laboratory, Department of Research, Changhua Christian Hospital, Changhua 500, Taiwan; 2 School of Big Data and Artificial Intelligence, Fujian Polytechnic Normal University, Fuqing 350300, China; 3 Department of Information Engineering and Computer Science, Feng Chia University, Taichung 407, Taiwanfrhsu@o365.fcu.edu.tw (F.-R.H.); 4 Cancer Research Center, Department of Research, Changhua Christian Hospital, Changhua 500, Taiwan; 5 Department of Animal Science and Biotechnology, Tunghai University, Taichung 407, Taiwan; 6 Comprehensive Breast Cancer Center, Changhua Christian Hospital, Changhua 500, Taiwan; 7 Department of Medical Image, Changhua Christian Hospital, Changhua 500, Taiwan; 8 Department of Medical Imaging, Chang Bing Show Chwan Memorial Hospital, Changhua 505, Taiwan

**Keywords:** breast cancer, surgical precision, cancer surgery, deep learning

## Abstract

Background: The assessment information of tumor margins is extremely important for the success of the breast cancer surgery and whether the patient undergoes a second operation. However, conducting surgical margin assessments is a time-consuming task that requires pathology-related skills and equipment, and often cannot be provided in a timely manner. To address this challenge, digital breast tomosynthesis technology was utilized to generate detailed cross-sectional images of the breast tissue and integrate deep learning algorithms for image segmentation, achieving an assessment of tumor margins during surgery. Methods: this study utilized post-operative tissue samples from 46 patients who underwent breast-conserving treatment, and generated image sets using digital breast tomosynthesis for the training and evaluation of deep learning models. Results: Deep learning algorithms effectively identifying the tumor area. They achieved a Mean Intersection over Union (MIoU) of 0.91, global accuracy of 99%, weighted IoU of 44%, precision of 98%, recall of 83%, F1 score of 89%, and dice coefficient of 93% on the training dataset; for the testing dataset, MIoU was at 83%, global accuracy at 97%, weighted IoU at 38%, precision at 87%, recall rate at 69%, F1 score at 76%, dice coefficient at 86%. Conclusions: The initial evaluation suggests that the deep learning-based image segmentation method is highly accurate in measuring breast tumor margins. This helps provide information related to tumor margins during surgery, and by using different datasets, this research method can also be applied to the surgical margin assessment of various types of tumors.

## 1. Introduction

Providing information on surgical margins during breast cancer surgery is crucial for the success of the operation. The margin is defined as the distance from the tumor to the cutting surface of the removed specimen. Currently, surgeons and radiation oncologists agree that no tumor should be left within 1 to 2 mm of the surgical specimen’s surface. If margins are still positive, there is a significant risk of tumor recurrence. This is particularly important for patients undergoing breast-conserving therapy (BCT) [1] for early-stage or ductal carcinoma in situ (DCIS), as it may lead to re-excision surgery. According to previous studies, about 25% of patients with invasive cancer and one-third of DCIS patients underwent re-excision surgeries [2,3].

Traditionally, determining whether cancer cells remain at the surgical margin is a collaborative effort between surgeons and pathologists. Surgeons are responsible for achieving sufficiently wide margins during surgery. Pathologists’ reports on clear margin widths vary based on the number of slices examined, which involves marking the margins with ink, including vertical incisions, scrapes, cavity edges, and compressing specimens for X-ray analysis. However, many hospitals do not perform intraoperative margin assessments for several reasons. The primary reason is that pathology-related techniques take a lot of time and need specialized expertise and equipment. Frozen section analysis, particularly for breast specimens, is technically challenging due to the high fat content that hampers effective freezing of the tissue [4,5]. Additionally, detecting tumor lesions near surgical margins (residual cells within <2 mm from the edge) is difficult, resulting in a higher rate of false negatives [6].

Several different strategies have been researched and applied in the past to address the issues mentioned above. For instance, methods utilizing Optical Coherence Tomography (OCT) and Deep Neural Networks (DNNs) for automatic edge evaluation of patient tissues have been explored. By using a dataset comprising 60 slices that include both healthy and tumorous tissue samples, these approaches achieved a sensitivity of 89% and specificity of 71% for cancer tissue classification [7]. Additionally, multimodal spectroscopic pathology that combines autofluorescence with Raman spectroscopy has been used to detect minute residual tumors on the surface of excised breast tissue [8]. However, a major issue with these studies is their reliance on specialized equipment such as OCT or Raman spectrometers, which are not commonly available in breast surgery operating rooms or radiology departments, thus limiting the practicality in clinical setting.

At this moment, we turn our attention to a technique known as Digital Breast Tomosynthesis (DBT). The effectiveness of mammography in detecting breast cancer varies; it is lower for women with heterogeneously dense or extremely dense breasts [9,10]. This is due to the fact that high breast density can obscure lesions (as overlapping tissues create a masking effect), or be misinterpreted as lesions due to the overlap of fibroglandular tissue [11]. DBT was developed to overcome the issue of tissue overlap by positioning the digital detector at specific angles relative to a pivot point and moving the X-ray source along an arc at predetermined ratios [12]. This allows for linear tomographic imaging of samples, with slight variations in detail depending on the imaging technique used. By employing algorithms for reconstructing images, it is possible to generate images of each tomographic plane. According to multiple retrospective studies, DBT has shown superiority or equivalence in observing areas of abnormality and microcalcifications compared to images produced by current full-field digital mammography (FFDM) systems [13,14]. Therefore, in recent years, the trend of replacing traditional FFDM with DBT imaging has begun to rise, significantly enhancing the clinical usability of such machines.

By leveraging DBT imaging’s ability to produce clear, unobstructed cross-sectional images of breast tissue and simultaneously utilizing the well-developed research on deep learning-based image region segmentation algorithms [15,16], we can effectively address this issue. This study explores the application of deep learning techniques to enhance the accuracy of tumor delineation in breast cancer surgery. The primary objective is to address the challenge of accurately identifying the boundary between malignant and healthy tissues, thereby facilitating more effective surgical procedures. The methodology used in this study comprises the utilization of a DBT image dataset with manually identified tumor areas. The results of the evaluation and performance metrics demonstrated the effectiveness of the methodology in enhancing the precision and efficiency of tumor region marking during breast cancer surgery [17,18].

## 2. Materials and Methods

**Figure 1:**
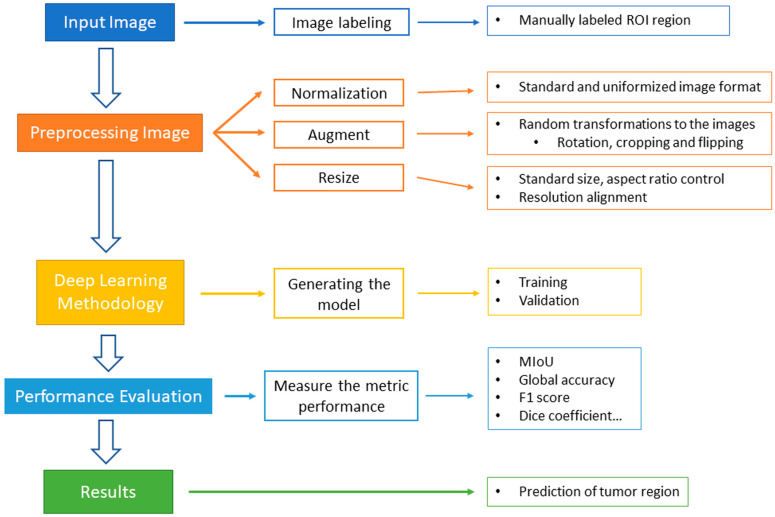
The overall workflow of this study.

The steps of this study include acquiring patient tissue images, preprocessing of tissue images, establishing a deep model (which includes dataset segmentation, model training and validation), and performance evaluation. The overall workflow of this manuscript has been illustrated in Figure 1. The details of the implementation at each stage will be elaborated on in the following paragraphs.

### 2.1. Image Data Collection Procedures

This study is a single-center, prospective study in which 50 patients who met the inclusion criteria and underwent breast-conserving surgery were randomly selected. The primary reason for selecting only 50 patients was due to the radiology department needing to prepare additional personnel to handle the imaging scans of tissue samples directly transmitted during surgery. After obtaining informed consent, post-operative tissue samples were sent to the radiology department for imaging collection before being forwarded to pathology. This study approved by the institutional review board (IRB) of Changhua Christian Hospital, Taiwan (No. 210624). Informed consent was collected, and the ethics committee reviewed all experimental methods to ensure conducted in accordance with the relevant guidelines and the Declaration of Helsinki. The inclusion criteria for this study were women aged 35–75 who were diagnosed with breast cancer at our hospital and underwent breast-conserving surgery from August 2021 to June 2023. Exclusion criteria include those who did not undergo breast-conserving surgery after diagnosis, or those who did undergo such surgery but had a tumor size of less than one centimeter. The pathological data of the organization were collected together to serve as the final standard for whether the tumor margins are positive and the actual size of the margins.

The imaging collection for patient tissues uses the Hologic Selenia^®^ Dimensions^®^ Mammography System (Hologic Inc., Marlborough, MA, USA). After the patient’s tissue was removed through breast-conserving surgery and sent to the radiology department, it was positioned on the DBT device according to three directions (12 o‘clock, 3 o‘clock, 6 o‘clock) pre-marked by the surgeon on the tissue for imaging. These organizations have been aseptically covered and uncompressed to prevent tissue damage or deformation. The height of each slice was set at 1 mm. Relevant aseptic and tissue transfer processes are conducted in accordance with the institution’s internal regulations. Due to the fact that the composition of DBT images is created by correcting the final image through X-rays shot from multiple angles, a two-centimeter diameter coin is placed as a scale and reference for image deformation while capturing tissue images. 

### 2.2. Image Data Preprocessing

To prepare the data, the first step is to remove extraneous elements unrelated to model learning, including directional markers and potentially distracting annotations, to ensure that images remain clear and suitable for evaluation. At the same time, image sizes are adjusted to a uniform dimension to meet the input requirements of deep learning models and avoid the impact of different resolutions and aspect ratios on diagnostic accuracy.

**Figure 2:**
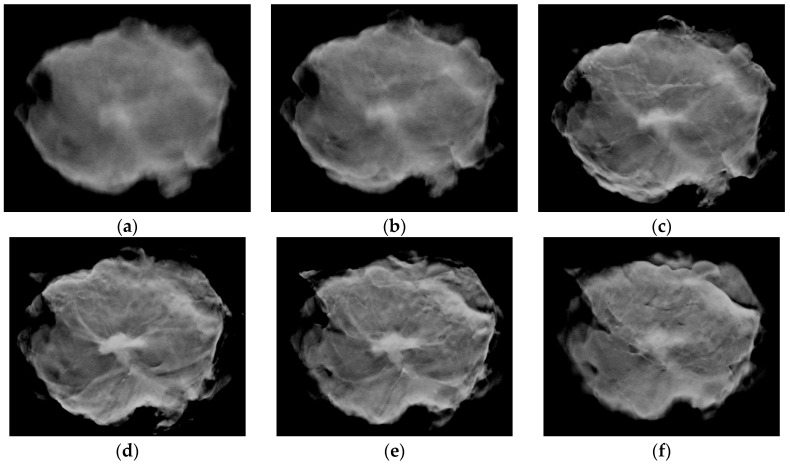
Images of the same tumor tissue presented on different sectional planes. This example dataset contains a total of 50 images. (**a**) The 1st slice, (**b**) the 7th slice, (**c**) the 14th slice, (**d**) the 21st slice, (**e**) the 28th slice, (**f**) the 35th slice.

Next, suitable images are selected from the DBT image dataset. As shown in Figure 2, within the sequence of images produced by DBT, those located at the beginning and end of the sequence tend to be more blurred. About 10–15 images situated in the middle of the sequence have the highest resolution and precise focus, allowing for clear differentiation between tumor tissues and normal tissues. We use manual inspection to select images from the DBT image sets of subjects that are suitable for training data. Depending on the actual size of the tumor, the actual number of images in the DBT image sets varies but, on average, about 10–15 images are selected for each subject.

**Figure 3:**
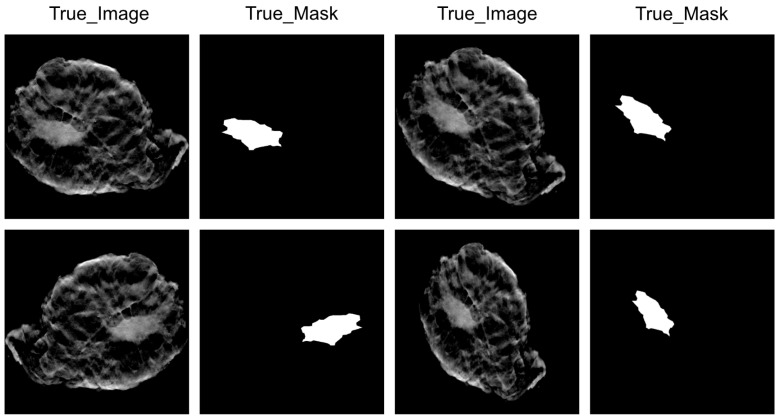
Masking of the tumor boundary. The column marked as “true_image” consists of original DBT images that display the internal characteristics of breast tissue. The column labeled as “true_mask” consists of corresponding mask images generated based on tumor areas manually annotated by experts.

For the selected images, the next step is to delineate the regions of interest (ROI) [19] based on surgical records, primarily depicting the image parts of tumor areas. This ensures that during model training, the algorithm focuses on relevant areas and improves the accuracy of tumor area labeling [20]. After extracting ROIs, generating the corresponding image masks completes the construction of the dataset. Data augmentation is also performed on the dataset, including stretching length and width at a fixed magnification and changing exposure among other methods to expand the sample size [21]. Figure 3 is a schematic diagram of some dataset images and the corresponding generated mask images.

### 2.3. Deep Learning Model

This study employs Unet3+ [22] as the deep learning model for identifying and segmenting tumor regions. UNet3+ is a deep learning algorithm and model for segmenting target images based on semi-supervised techniques. The U-Net3+ architecture makes significant improvements over the original U-Net model [23], primarily through the use of an enhanced U-shaped pyramid-dilated network algorithm. By training the UNet3+ network on annotated datasets, it establishes a model capable of detecting specific image features and providing segmented areas. The training process includes iteratively adjusting network parameters through appropriate optimization algorithms and minimizing selected loss functions to enhance accuracy. By incorporating full-scale skip connections, deep supervision, and dense connections into the decoder, it becomes suitable for complex image segmentation tasks. Full-scale skip connections are crucial for merging multi-scale feature maps from various stages of the network, ensuring efficient use of both high-level and low-level features.

**Figure 4:**
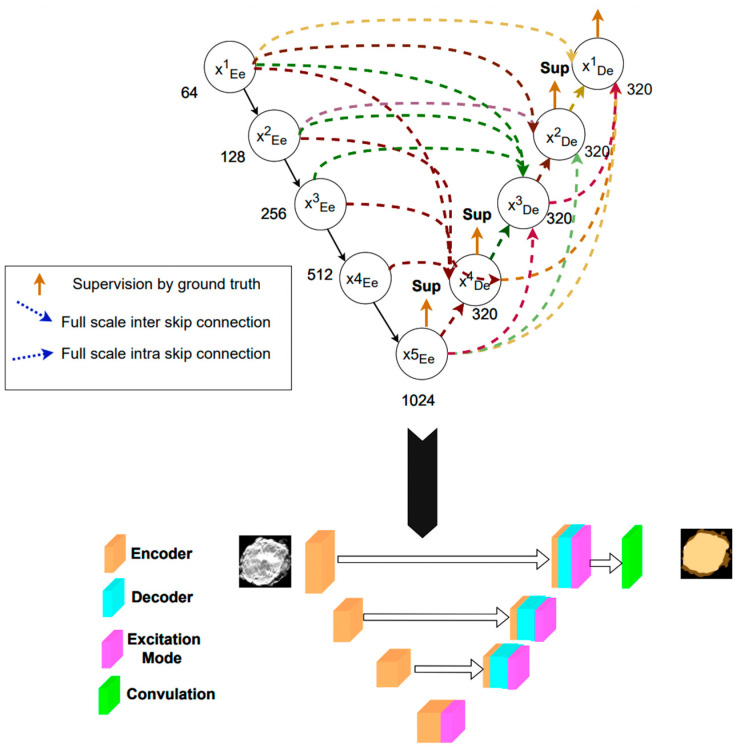
Unet3+ algorithm framework. The direction of the dashed arrows indicates how Unet3+ integrates feature maps of different sizes at each encoder layer through skip connections.

Figure 4 is the network architecture diagram of Unet3+. Internally in U-Net3+, directly comparing differences between intermediate network outputs and real situations at multiple levels plays a significant role in promoting gradient flow and significantly improving segmentation accuracy [24]. It also employs max pooling to reduce the size of the feature maps, ultimately achieving a full-size feature fusion. This characteristic is not possessed by Unet. According to the direction of the dashed lines, we can find that Unet3+ integrates feature maps of different sizes at every encoder layer through the use of full-size skip connections. This feature harmonization allows the network to fully perceive the visual environment across multiple scales, which is essential for accurately delineating boundaries.

### 2.4. Training Protocol and Infrastructure

The computational environment used in this study is established on a virtual machine allocated by a virtual computing platform. The allocated virtual machine includes an 8-core virtual CPU (provided by Intel^®^ Xeon^®^ Gold Series 61 processors) (Intel, Santa Clara, CA, USA), 100 GB of virtual hard disk, 90 GB of virtual memory, and an NVIDIA^®^ Tesla V100 GPU (NVIDIA, Santa Clara, CA, USA) with 32 GB of video RAM assigned in physical form. The operating system used is Ubuntu 20.04 LTS. The graphics processing unit accelerated computing environment was built using NVIDIA Compute Unified Device Architecture (CUDA) version 12.2 and the NVIDIA CUDA Deep Neural Network library version 8.9.2.26.

Image enhancement techniques were applied to the dataset to improve model adaptability; these include random scaling (from 0.8× to 1.2×), rotation (from −90° to +90°), cropping, vertical/horizontal flipping, and elastic deformation. The epochs are set to 100, batch size is set to 6, and the learning rate (lr) is 3 × 10^−4^. After argumentation, the dataset is divided into a training set, validation set, and testing set in a ratio of 7:3:1 for training and validation.

Deep learning programs and performance evaluation metric programs in this study were implemented using Python 3.6 (Python Software Foundation) and PyTorch 2.0 [25]. Relevant learnable parameters (such as weights and biases) are also stored in the model file. By using the built-in functions of the PyTorch framework, the trained model can be read and load easily and applied for inference purposes.

### 2.5. Performance Evaluation

In this study, the trained model was evaluated by using ground truth images data set masked manually to find the Mean Intersection over Union (MIoU). This computes the average intersection over union (IoU) scores for all classes, as global accuracy. It is usually computed as the proportion of correctly categorized pixels to the total number of pixels in image dataset. Precision evaluates a model’s ability to make accurate positive predictions. It is defined as the ratio of true positive predictions to the overall number of positive predictions (including true positives and false positives).

Recall (also known as sensitivity) evaluates a model’s ability to find all relevant instances in a dataset. It is defined as the ratio of genuine positive predictions to total positives (true positives and false negatives combined). The F1 score is the harmonic mean of precision and recall, resulting in a single statistic that balances a model’s precision and recall, which is especially beneficial when class label distribution is unequal. The F1 score has its highest value at 1 (perfect precision and recall) and lowest at 0. The dice coefficient measures the overlap between two samples. It is frequently used to evaluate the effectiveness of picture segmentation techniques. The dice coefficient is comparable to the F1 score, except it is used to assess the similarity of two samples. It ranges from zero (no overlap) to one (complete overlap) [26].

## 3. Results

**Figure 5:**
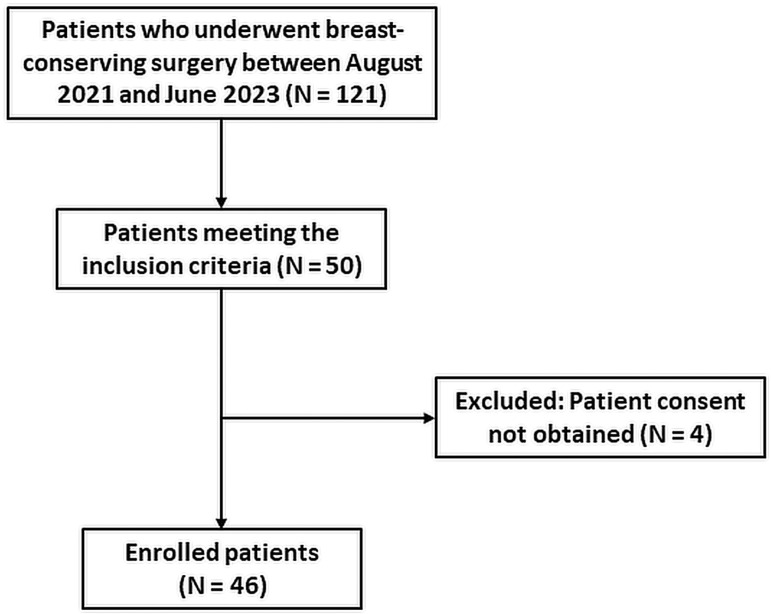
The process of patient inclusion and exclusion in this study.

After screening 121 patients who underwent breast-conserving surgery from August 2021 to June 2023, we randomly selected 50 patients who met the inclusion criteria and obtained tissue samples and informed consent. Four patients were excluded from this study because informed consent could not be obtained. In the end, we obtained 48 DBT image sequence data generated from tissue samples of 46 patients who underwent breast-conserving surgery. Although receiving neoadjuvant chemotherapy was not an exclusion criterion for this study, none of the 46 patients received neoadjuvant chemotherapy. The process of patient inclusion and exclusion in this study is shown in Figure 5.

In terms of clinical characteristics, the average size of the maximum diameter of all excised tissue specimens is 6.01 cm. Based on the actual size differences of the tumors excised from each patient, the number of slices in each imaging sequence ranges from about 40 to 60. Following the descriptions in previous research methods, for each patient, 10 to 15 slices with clear focus and tissue contours are selected for ROI extraction. Pathological data indicate that the average widths of the tumor margins in four directions (3‘, 6‘, 9‘, and 12’ o‘clock) are 1.3 cm, 1.03 cm, 1.15 cm, and 1.29 cm, respectively. There are five patients with a margin of resection less than or equal to 0.2 cm in any one direction. Table 1 lists all the clinical characteristics of the enrolled patients, including clinical staging data, lymph node metastasis status, and tumor size information. The tumor size is reported based on the results of the pathology report.

After these slice images were augmented, a total of 1292 images were obtained. We divided them into a training dataset (containing 1140 images) and a test dataset (containing 152 images), where the training dataset was used for model training and validation. On the training data set: Mean Intersection over Union (MIoU) 0.91, global accuracy 0.99, precision: 0.98, recall: 0.83, F1 score: 0.89, dice coefficient: 0.93. On the test set, the number of images is 152; Mean Intersection over Union (MIoU): 0.83, global accuracy: 0.97, precision: 0.87, recall: 0.69, F1 score: 0.76, dice coefficient: 0.86. Table 2 shows the training outcomes and performance of the model on both the training dataset and test dataset.

**Figure 6:**
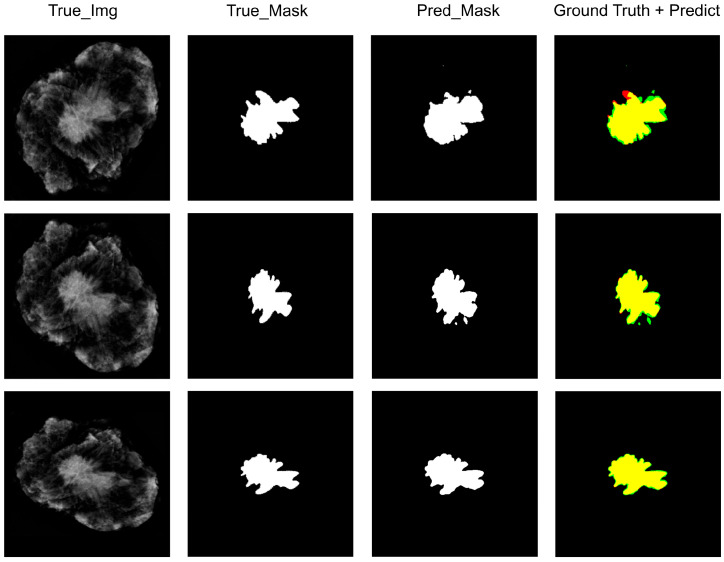
Algorithm Unet3+ with true image, true mask, ground truth + predict & prediction mask.

As shown in Figure 6, the first column, labeled ‘true_img’, contains the original DBT images that reveal the internal features of breast tissue. The second column, ‘true_mask’, presents manually annotated ground truth data by experts outlining the tumor area within each image. These masks serve as a benchmark for identifying tumor locations. The “Ground truth + predict” column displays an overlay of model predictions on top of the ground truth, where intersections appear in a mixed hue (green, in this case), allowing for a visual assessment of prediction accuracy against actual data. The final column shows predicted masks generated by Unet3+, depicted as binary images with white areas representing predicted regions of interest. Based on the three randomly selected slice images shown in Figure 6, along with their corresponding ground truth and the predicted masks, except for a small discrepancy in the tumor boundary of the first image (indicated in red) from the ground truth, the other four images almost perfectly align with the ground truth. This demonstrates that the trained Unet3+ model has an exceptional ability to detect tumor boundaries.

**Figure 7:**
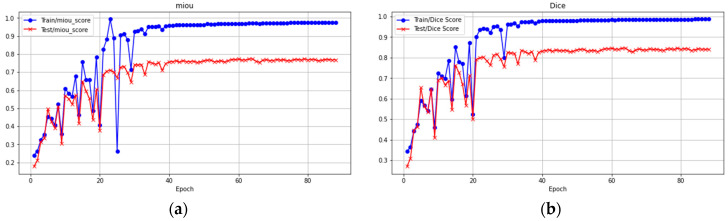
The performance comparisons based on (**a**) MIoU score and (**b**) dice score.

As shown in Figure 7, the blue curve represents the training score, while the orange curve depicts the test score, including both MIoU and dice scores. The MUoU training score starts high, but experiences a sharp decline during the first epoch, continuing until around epoch 20. This indicates rapid learning from the training data. After an initial steep decrease, the test score begins to mirror the training score; however, it levels off after 21 epochs, suggesting that model performance on test data has stabilized. For dice scores, both training and test scores follow a similar trend until after 25 epochs when the training score increases as the test score plateaus. In both cases, test loss eventually converges with training loss, demonstrating good model generalization to new and unseen data.

## 4. Discussion

Due to the fact that this type of research currently belongs to a relatively novel field, there is not much prior literature available for direct comparison, and there are differences in the methodology as well. Therefore, comparisons regarding efficacy should only be used as references. A recent review article thoroughly explores the initial progress of various technologies implemented in real-time margin assessment during breast-conserving surgery in recent years, including the use of OCT, specific fluorescent tissue staining, ultrasound, and traditional radiographic histology imaging [27]. Using OCT imaging for positive and negative margin determination, its specificity is reported in the literature to range from approximately 82% [28]. A meta-analysis on OCT-related research, which combined 18 studies and samples from 782 patients, estimated the specificity of detecting margins to be as high as 0.88. However, the OCT technologies utilized in this study encompass four less common types of OCTs that claim high resolution and are currently less available (FF-OCT, UHR-OCT, SS-OCT, and PS-OCT), thus cannot be directly compared the performance with the recently OCT-related research [29]. Imaging using Raman spectroscopy combined with sampling and data algorithms (also named multimodal spectral pathology) has a sensitivity and specificity of approximately 95% and 82%, respectively [8]. Using ultrasound imaging combined with deep learning for margin assessment, the prior literature indicates its specificity is about 76% [30].

This study does not directly judge the positive or negative margins; instead, by combining DBT images that provide multiple slices of a single tissue and the ability of deep models to accurately delineate tumor margins in images, using trained models to infer and outline the edges on DBT images of excised tissues allows for an intuitive understanding of whether the margin depth is sufficient. The extraction of areas of interest is conducted with pathology reports and circled by experienced physicians to ensure that algorithms focus on key areas, thereby improving the accuracy of tumor area markings. In our training and testing results, the MIoU of up to 0.83 and a dice coefficient of 0.86 were achieved in the test dataset, indicating high congruence between this model and actual tumor areas. The methodology of this study holds promise for improving the precision and efficiency of margin determination during breast-conserving surgery.

**Figure 8:**
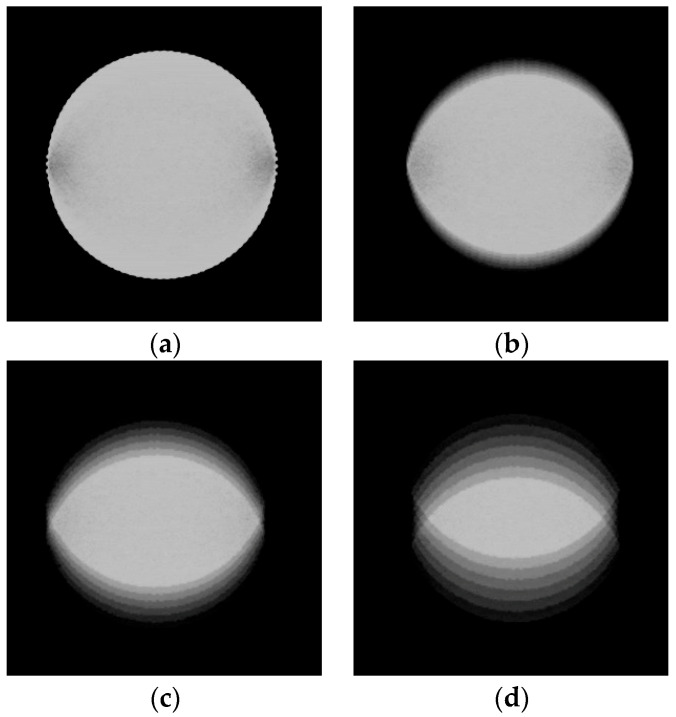
Schematic diagram of the deformation effects on slices at different positions in the DBT image sequence. This example sequence consists of 50 images. (**a**) Slice 1, (**b**) slice 17, (**c**) slice 32, (**d**) slice 47.

This study still has areas for future improvement, which have been outlined as our next steps. Firstly, the determination of margins in this research is based on physicians’ experience and radiographic presentation to depict optimal outcomes, without the ability to judge whether sample images’ margins are positive or negative. It also does not assess if the actual margin is sufficient (e.g., greater than 0.2 cm), due to several technical challenges that are currently insurmountable. One such challenge arises from the imaging principle of breast tomosynthesis, where images in the middle of a sequence exhibit more severe distortion compared to those at the beginning (closest to the first image) which show minimal deformation, and those at the end (closest to the last image) which display maximum distortion. Typically, images with clearest focus are located in the middle of a sequence; hence it is impossible to avoid issues related to deformation. Figure 8 demonstrates this with a random patient’s imagery (a total of 50 tomographic images produced); by cropping the scale parts from images at different positions in the sequence and viewing them together, one can intuitively observe the differences in how the images are affected by deformation. In Figure 8a (at position one in an image sequence), it can be observed that while the coin appears nearly circular while in Figure 8b (position 17) and Figure 8c (position 32), its shape distorts into an ellipse due to compression. Figure 8d, located at the end of the sequence (at position 47), is the image most severely affected by the deformation. Although there are algorithms designed for correcting distorted imagery today [31,32], they involve modifying original images, making it uncertain whether compensated images accurately reflect true tumor margin widths; thus, we will not delve further into this matter here.

In terms of determining the margins between positive and negative results, the current best practices still rely on pathological staining of cells about 2 mm from the margin surface for determination, and merely using image features for analysis presents its challenges. Regardless, these open questions provide us with ideas for further improvements and advancements in this research area. Nonetheless, these open questions provide valuable insights for refining and advancing our research further.

Artificial intelligence technology delivers precise and sensitive image analysis results, greatly benefiting breast cancer management. Currently, many studies have applied AI in various aspects of breast cancer and even other cancers’ management [33,34,35], including digital pathology [36], clinical prognosis, and biopsy result analysis, among others. It is worth mentioning that although the imaging source used in this study is based on DBT images of breast cancer tumor tissues, the principle of determining margins is not unique to breast cancer. This also suggests that, for surgeries involving the removal of solid tumors with positive margin concerns, like ovarian and prostate cancers [37], leveraging tissue image samples from various cancers to develop deep learning models could extend this research to additional cancer types. This approach could also aid in intraoperative margin evaluations for other cancers. However, this will also require further exploration into different types of tumor images and imaging technologies in the future.

## 5. Conclusions

The preliminary assessment results show that the deep learning-based image segmentation method proposed in this paper has high accuracy in positioning, delineating, and measuring breast tumor margins. It reduces the uncertainty in surgical procedures, and has strong potential in clinical applications. The combination of this method with digital breast tomosynthesis can further improve the accuracy and stability of the margin delineation, which is worth exploring in future work.

## Figures and Tables

**Table 1:** Characteristics of all enrolled patients.

Characteristics	Total (*n* = 46)
Pathology	
IDC	32
ILC	4
DCIS/LCIS	6
MUC	4
Lymph node status	
Negative	12
Positive	34
Histologic grade	
1/2	43
≥3	3
Tumor Size	
≥2 cm	32
1 to 2 cm	11
≤1 cm	3

IDC: invasive ductal carcinoma, ILC: invasive lobular carcinoma, DCIS: ductal carcinoma in situ, LCIS: lobular carcinoma in situ, MUC: mucinous carcinoma.

**Table 2:** The performance comparison is based on training and test data set.

U-Net3+	Training Dataset	Test Dataset
Mean Intersection over Union (MIoU)	0.91	0.83
Global accuracy	0.99	0.97
Precision	0.98	0.87
Recall	0.83	0.69
F1 score	0.89	0.76
Dice coefficient	0.93	0.86
Data size	1140	152

## Data Availability

The datasets produced and analyzed in this study are not publicly accessible due to IRB and institutional limitations.
